# Kinlessness, Loneliness, and End of Life: A Cross-National Comparison of 20 Countries

**DOI:** 10.1093/geroni/igaa057.2039

**Published:** 2020-12-16

**Authors:** Esteban Calvo, Christine Mair, Katherine Ornstein, Rosario Donoso, José Medina

**Affiliations:** 1 Universidad Mayor, Santiago, Region Metropolitana, Chile; 2 University of Maryland, Baltimore County, Baltimore, Maryland, United States; 3 Icahn School of Medicine at Mount Sinai, New York, New York, United States

## Abstract

Countries across the globe are experiencing declining rates of fertility and marriage, which present a distinct challenge for older adults’ social integration, well-being, and end-of-life care. However, older adults who are “alone” (e.g., no partner, no child) may not be lonely, and end-of-life risks faced by “kinless” older adults likely vary significantly by country context. Using harmonized, cross-national data from 20 countries (United States (HRS), England (ELSA), and European Union (SHARE)), we examine associations between family structure, loneliness, and end-of-life outcomes. Although “kinless” family structures are associated with greater loneliness in the pooled sample, the percent of “kinless” who report no signs of loneliness ranges from 7% (Greece) to 56% (Denmark). Family structure is associated with various end-of-life outcomes, and these associations vary by country—likely reflecting differences in healthcare structure. We discuss distinctions between “being alone,” “being lonely,” and “being without care” in light of cross-national variation.

